# Validation and comparison of the stroke prognosis instrument (SPI-II) and the essen stroke risk score (ESRS) in predicting stroke recurrence in Asian population

**DOI:** 10.1186/s12883-023-03329-w

**Published:** 2023-08-01

**Authors:** Junporn Kongwatcharapong, Akaporn Sornkhamphan, Chitapa Kaveeta, Surakit Nathisuwan

**Affiliations:** 1 Pharmaceutical Care in Inpatient unit, Department of Pharmacy, Siriraj Hospital, Bangkok, Thailand; 2 Division of Neurology, Department of Medicine, Faculty of Medicine Siriraj Hospital, Mahidol University, Bangkok, Thailand; 3 Clinical Pharmacy Division, Department of Pharmacy, Faculty of Pharmacy, Mahidol University, 447 Sri-Ayutthaya Road, Ratchathewi, Bangkok, 10400 Thailand

**Keywords:** ESRS scores, SPI-II scores, Recurrent stroke

## Abstract

**Background:**

Currently, there are limited data on the accuracy of available risk scores to predict stroke recurrence in the Asian population.

**Method:**

A single-center, retrospective cohort study was conducted among patients with acute ischemic stroke during January 2014 - December 2018. Longitudinal data with three years of follow-up among these patients were collected and validated through both electronic and manual chart review. The area under the receiver-operating curve (AUROC) method or C-statistic and calibration plot were used to evaluate and compare the Stroke Prognosis Instrument II (SPI-II) and the Essen Stroke Risk Score (ESRS) in predicting the long-term risk of recurrent ischemic stroke. The predictive performances of the two scores were compared using DeLong’s method.

**Results:**

The study cohort consisted of 543 patients, including 181 and 362 patients with and without recurrent events. There were no significant differences in mean age and gender between the two groups. Recurrence cases tended to have significant more risk factors compared to those without events. Among cases with recurrent events, 134 (74.03%) and 65.74% (119) cases were classified as high-risk based on SPI-II and ESRS, respectively. The AUROC curve of the SPI-II and ESRS score was 0.646 (95% CI, 0.594–0.697) and 0.614 (95%CI, 0.563–0.665), respectively (p = 0.394). Based on the calibration plot, the SPI-II and ESRS scores showed similar moderate predictive performance on recurrence stroke with a C statistic (95% CI) of 0.655 (95% CI: 0.603–0.707) and 0.631 (95% CI 0.579–0.684), respectively.

**Conclusion:**

Both ESRS and SPI-II scores had moderate predictive performance in Thai population.

**What is already known about this subject**:

- Currently, there is limited data regarding the accuracy of risk score to predict stroke recurrence in Asian population.

**What this study adds**:

- The Stroke Prognosis Instrument (SPI-II) and the Essen Stroke Risk Score (ESRS), a commonly used risk score, developed based on Western population cohort, possessed moderate predictability in predicting long-term stroke recurrence in Asian population.

## Introduction

Stroke is among the leading causes of death and disability in low and middle-income countries (LMIC) [1]. Thailand is a middle income country in Southeast Asia with a population of 65 million. Data from Thailand’s national health statistic indicated that there were more than 355,000 stroke cases in the year 2019 [2]. Stroke was also the major contributor to disability adjusted life years (DALYs) lost in 2017 globally [3]. Despite such a magnitude of the problem, stroke care remains poor, especially in developing countries, partly due to a less advanced health system and suboptimal public health literacy of the public [4]. As a result, stroke recurrence seems to be much higher in developing countries or less mature health systems compared to developed countries [5, 6]. A recent study in China showed that 41% of stroke survivors had recurrent stroke at 5 years while data from a recent registry of developed countries showed only 9.5% of recurrence in 5 years [7, 8]. With multiple recurrence, mortality and disability rates are therefore much higher in developing countries [1, 3].

To better predict risk of future recurrence, a number of risk scores have been developed to assist clinicians in identifying patients who are at high risk of stroke recurrence [9]. Among these risk scores, the Stroke Prognosis Instrument II (SPI-II)[10] and the Essen Stroke Risk Score (ESRS)[11] have been developed and validated in various populations showing potential utility in a diverse group of patient population. These two scores are commonly used due to their relative ease of use and readily available variables for the models. These risk scores however were developed in mostly Caucasian population and in developed healthcare systems. Previously, both SPI-II and ESRS scores have been evaluated and compared on the predictive accuracy in Asian population [12, 13]. Results of these studies indicated that these scores had moderate predictive performance in Asian population. However, little data exists on the predictive performance of these risk scores in developing Southeast Asian countries including Thailand. Therefore, this study aimed to evaluate and compare the predictive performance of SPI-II and ESRS scores in predicting long-term stroke recurrence among Thai ischemic stroke survivors. The results of this study may provide useful information on the possibility of adopting these risk scores to identify and streamline limited healthcare resources to care for patients at high risk of recurrence.

## Methods

### Study design and setting

A retrospective observational study was conducted among patients who experienced and survived acute ischemic stroke during January 2014 - December 2018. The study site was the Siriraj Hospital, a 2,500 bed, university-affiliated, tertiary care hospital in Bangkok, Thailand. The hospital is equipped with a 17-bed stroke unit. There were 21 full-time staff neurologists with board certification in neurology who provided care for patients under the supervision of the Neurology Department. In addition, the hospital embraces multidisciplinary approach in stroke care with a multidisciplinary team of physicians, nurses, pharmacists and other allied health workers who work together both for outpatient and acute care settings. The study was approved by the Institutional Review Board of Siriraj Hospital (IRB Number: Si642/2019, date of approval: September 17, 2019) and followed the principles of the Declaration of Helsinki. The informed consent of patients was waived by the Institutional Review Board of Siriraj Hospital due to the retrospective nature of the study.

### Study population

All adult patients who experienced and survived acute ischemic stroke (International Classification of Disease Tenth Revision or ICD-10 of 1630, 1631, 1632, 1633, 1635, 1638 and 1639) during January 2014 - December 2018 were screened. Patients who had regular follow-up for at least three years at Siriraj Hospital were included. Exclusion criteria included (1) cerebral embolism, (2) presence of atrial fibrillation, (3) missing critical data for risk score calculation, (4) referral patients with-out longitudinal follow-up information, and (5) loss to follow-up. Two-step approaches were used to identify patients including case identification by the ICD‐10 and manual chart review for case confirmation and data collection.

### Data collection

Data were collected manually from medical chart and hospital database where appropriate and transferred into a standardized, case record form by two clinical pharmacists. Demographic data including age, gender, smoking status and alcohol use were collected. Comorbidities were obtained from clinical diagnosis from the medical chart and/or the International Classification of Diseases 10th Revision (ICD-10) coding from the hospital database. Stroke related information consisted of types of stroke, duration of stroke, level of disability were also collected. All cases of stroke were confirmed by computed tomography (CT) and/or magnetic resonance imaging (MRI).

### Risk scores and recurrent stroke

Details on the components and weight of each component for both the SPI-II and ESRS scores are shown in Table 1. Generally, while both scores used age and presence of risk factors and comorbidities, the differences are the presence (and absence) of some variables, including smoking, stroke, peripheral arterial disease and heart failure as variables [10, 11]. There are also differences in the weighting of common variables in the scores. In addition, these scores used different risk categories. SPI-II classifies patients into low (0–3 points), moderate (4–7 points), and high risk (8–15 points) groups, while ESRS classifies patients into either low (0–2 points) or high risk (3–9 points) groups. These differences reflect different study designs, study population and the methods used to construct the models and identify risk thresholds [10, 11]. For recurrent events, two-step approaches were used to identify patients with stroke recurrence including case identification by ICD‐10 and manual chart review for case confirmation.

**Table 1:** List and weighting of variables in the Stroke Prognosis Instrument II (SPI-II) and Essen Stroke Risk Score (ESRS)

Risk factor	Scale	
	SPI-II	ESRS
Age < 65 years	0	0
Age 65–70 years	0	1
Age 70–75 years	2	1
Age > 75 years	2	2
Hypertension	1	1
Diabetes mellitus	3	1
Smoking	-	1
Prior cerebral infarction or transient ischemic attack (TIA)	3^†^	1
Prior myocardial infarction	1^‡^	1
Stroke (not TIA)	2	-
Peripheral arterial disease	-	1
Other cardiovascular diseases (except atrial fibrillation and myocardial infarction)	1^‡^	1
Congestive heart failure	3	-
**Total scores**	15	9
**Low**	0–3	0–2
**Moderate**	4–7	
**High**	8–15	3–9

† Refers to prior cerebral infarction but not prior TIA on the SPI-II scale.

‡ In the SPI-II scales, coronary artery disease was scored with one point. For example, a patient who suffered myocardial infarction before and now is suffering angina will be scored one point by SPI-II scale but two points by ESRS scale.

### Statistical analysis

Continuous variables were reported as numbers and percentages and calculated as the mean ± standard deviation (SD) or median (interquartile range, IQR) depending on the distribution detected by normality tests. Stroke outcomes by the ESRS and SPI-II scores were computed as the rate of events per 100 patient-years. Independent two-proportion sample were compared using either the chi-square or Fisher’s exact test as appropriate. The significance of continuous variable as a two-independent sample was assessed with Independent t-test or Mann Whitney U test following normality assumption. The ROC curve was performed by discriminant threshold for the ESRS and SPI-II score to obtain the optimum values with the highest sensitivity and specificity compared to stroke recurrence. Cox proportional hazards models were used to determine the risk for each threshold through the hazard ratio and the 95% confidence interval (CI), respectively. The area under the receiver operating curve (AUROC) method or C-statistics was used to test the diagnostic accuracy for stroke risk classification of ESRS and SPI-II scores. The predictive performances of the two scores were compared using DeLong’s method [14]. In addition, the calibration plots with score of 0, 1, 2, 3, ≥ 4 for both ESRS and SPI-II was evaluated. Statistical significance was defined as p < 0.05. All statistical analyses were performed by using SPSS version 18.0 and STATA version 14.1.

## Results

There were a total of 543 patients who met the inclusion criteria, 181 patients had recurrent stroke during the follow-up period. The baseline characteristics of the study population are shown in Table 2. Mean ages of those with and without recurrent stroke were not significant different (65.95 $\pm$ 13.66 vs. 65.41 $\pm$ 13.81 years for those with recurrent stroke and those without recurrent events, p = 0.667). No difference in gender distribution existed. For stroke subtypes, 46.4%, 24.9% and 28.7% were large vessel disease, small vessel disease and stroke of undetermined etiology, respectively. A history of prior stroke or TIA was more common in patients with recurrent events compared to those without recurrent events. In addition, patients with recurrence tended to have higher rates of cardiovascular risk factors including hypertension, impaired fasting glucose, peripheral arterial disease, prior myocardial infarction, and other cardiovascular diseases. Among 181 cases with recurrent events, 134 (74.03%) and 65.74% (119) cases were classified as high-risk based on the SPI-II and ESRS, respectively. Risk of recurrent stroke after ischemic stroke stratified by Stroke Prognosis Instrument II (SPI-II) and Essen Stroke Risk Score (ESRS) are shown in Table 3.

**Table 2:** Baseline characteristics of the study cohort, separated and compared between patients with and without recurrent stroke

Characteristics	Patients without stroke recurrence N = 362 (%)	Patients with stroke recurrence N = 181 (%)	P value
Age			
Mean$\pm$ sd (years) (min-max)	65.41 $\pm$ 13.81 (25–99)	65.95 $\pm$ 13.66 (28–93)	0.667
< 65 years	164 (45.3)	73 (40.3)	0.800
65–70 years	32 (8.8)	30 (16.6)	
70–75 years	60 (16.6)	31 (17.1)	
> 75 years	96 (26.5)	47 (26.0)	
Sex			
Male	174 (48)	98 (54)	0.182
Cardiovascular risk factors			
Hypertension	278 (76.8)	155 (85.6)	0.016
Diabetes mellitus	152 (42.0)	79 (43.6)	0.713
Impaired fasting glucose	8 (2.2)	12 (6.6)	0.010
Prior cerebral infarction	0	54 (29.8)	0.000
Prior TIA	5 (1.4)	14 (7.7)	
Hyperlipidemia	210 (58.0)	119 (65.7)	0.082
Coronary heart disease	23 (6.3)	25 (13.8)	0.082
Peripheral arterial disease	1 (0.3)	4 (2.2)	0.045
Prior myocardial infarction	21 (5.8)	20 (11)	0.038
Other cardiovascular disease (except AF/MI)	12 (3.3)	14 (7.7)	0.023
Heart failure	5 (1.4)	6 (3.3)	0.193
CKD Stage 3 Stage 4 Stage 5	16 (57.1) 5 (17.9) 7 (25.0)	18 (75.0) 2 (8.3) 4 (16.7)	0.459
Smoking	113 (31.2)	48 (26.5)	0.259
Alcohol	98 (27.1)	49 (27.1)	1.000

**Table 3:** Risk of recurrent stroke after ischemic stroke stratified by Stroke Prognosis Instrument II (SPI-II) and Essen Stroke Risk Score (ESRS).

SPI-II Score	Patients (%)	Strokes (%)	Percent risk (95% CI)
0			
1	2 (0.4%)	2 (1.1%)	100 (15.8–100.0)
2	64 (11.8%)	16 (8.8%)	25.0 (15.0–37.4)
3	102 (18.8%)	29 (16.0%)	28.4 (19.9–38.2)
4	28 (5.2%)	6.0 (3.3%)	21.4 (8.8–40.9)
5	92 (16.9%)	21 (11.6%)	22.8 (14.7–32.7)
6	107 (19.7%)	34 (18.8%)	31.8 (23.1–41.5)
7	10 (1.8%)	4 (2.2%)	40 (12.6–73.8)
8	88 (16.2%)	32 (17.7%)	36.4 (26.4–47.3)
9	32 (5.9)	21 (11.6)	65.6 (46.8–81.4)
10	2 (0.4)	2 (1.1)	100 (15.8–100.0)
11	9 (1.7)	8 (4.4)	88.9 (51.7–99.72)
12	7 (1.3)	6 (3.3)	85.7 (42.1–99.6)
13	0	0	0
14	0	0	0
15	0	0	0
Total	543 (100%)	181 (100%)	
ESRS score	Patients (%)	Stroke (%)	Percent risk (95% CI)
0	35 (6.4%)	6 (3.3%)	17.1 (6.6–33.7)
1	86 (15.8%)	25 (13.8%)	29.1 (19.8–39.9)
2	135 (24.9%)	31 (17.1%)	22.9 (16.2–30.9)
3	151 (27.8%)	52 (28.7%)	34.4 (26.9–42.6)
4	91 (16.8%)	43 (23.8%)	47.3 (36.7–58.0)
5	35 (6.4%)	17 (9.4%)	48.6 (31.4–66.0)
6	9 (1.7%)	6 (3.3%)	66.7 (29.9–92.5)
7	1 (0.2%)	1 (0.6%)	100 (2.5–100.0)
8	0	0	0
9	0	0	0
Total	543 (100%)	181 (100%)	

### Comparison of SPI-II vs. ESRS scores

**Fig. 1:**
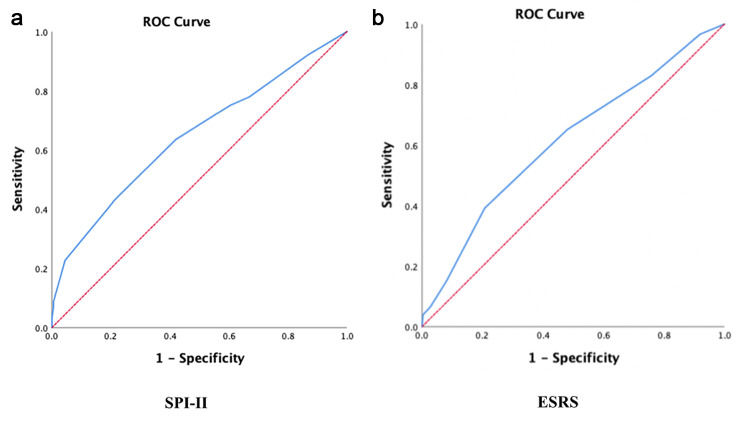
The receiver operating characteristic (ROC) curve in predicting recurrent stroke of **(A)** the Stroke Prognosis Instrument II or SPI-II and **(B)** the Essen Stroke Risk Score or ESRS

Overall, both the SPI-II and ESRS scores showed moderate predictive performance in recurrent stroke with AUROC (95% confidence interval [CI]) of 0.646 (0.594–0.697) and 0.614 (0.563–0.665) as shown in Fig. 1(A) and Fig. 1(B), respectively. There was no statistical significance between the two scores (p = 0.394). SPI-II score of ≥ 4 showed 63% sensitivity and 58% specificity with positive predictive value (PPV) of 43% (95% CI: 39.07–47.09) and negative predictive value (NPV) of 76% (95% CI: 72.06–79.74). ESRS score of > 3 showed 65% sensitivity and 51% specificity with PPV of 40% (95% CI: 36.80-44.06) and NPV of 75% (95% CI: 70.52–78.88).

**Fig. 2:**
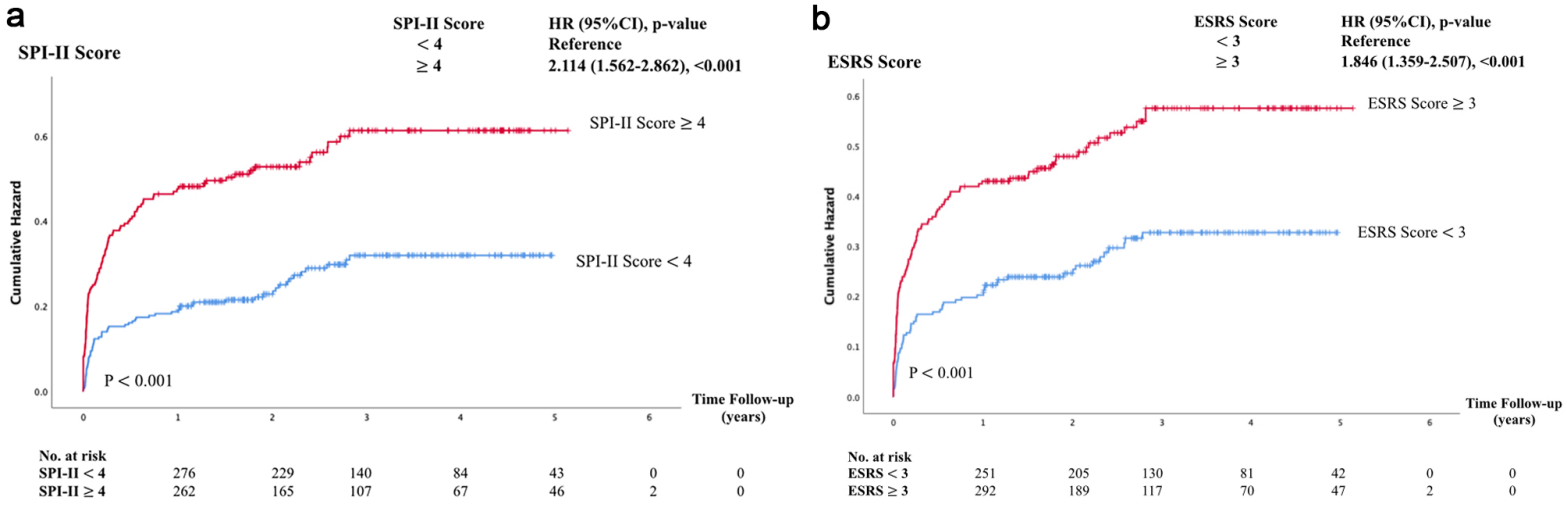
Cumulative incidence of recurrent stroke for **(A)** The Stroke Prognosis Instrument II or SPI-II and **(B)**the Essen Stroke Risk Score or ESRS

The results of the Cox proportional hazards models showed that patients with a SPI-II score of ≥ 4 (high risk group) had a significantly higher risk of recurrent events compared to those with a SPI-II score of < 4 (HR: 2.12; 95% CI: 1.562–2.862, p < 0.001) as shown in Fig. 2(A). Patients with an ESRS score of > 3 (high-risk group) also had a significantly higher risk of recurrent events compared to those with an ESRS score of < 3 (HR: 1.85; 95% CI: 1.359–2.507, p < 0.001), as shown in Fig. 2(B) The risk of a recurrent event significantly increased at the beginning at one year after the index event.

### Predictive performance of SPI-II and ESRS scores

**Fig. 3:**
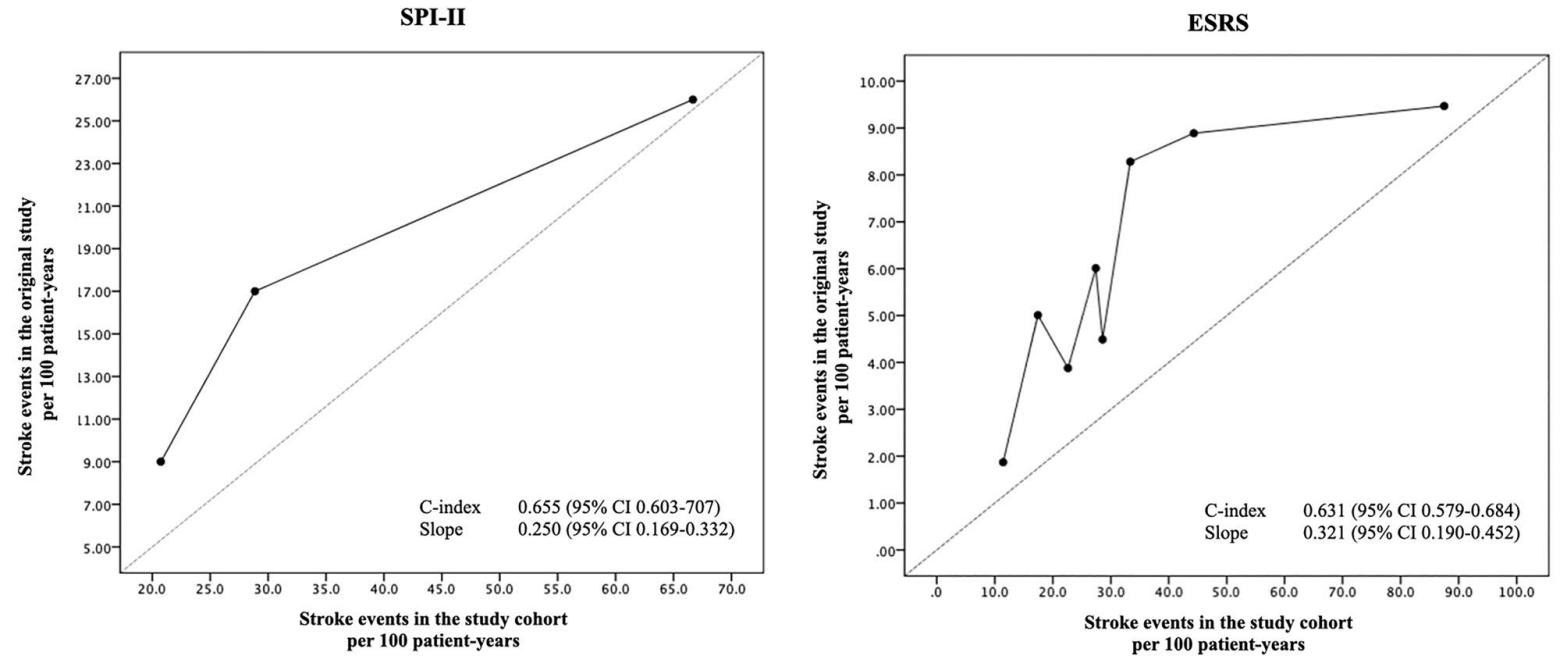
Calibration plot of **(A)** the Stroke Prognosis Instrument II or SPI-II and **(B)** Essen Stroke Risk Score or ESRS

The predictive performance of the SPI-II and ESRS scores was evaluated using calibration plots. The C index and the slope of the SPI-II score were 0.655 (95% CI: 0.603–0.707) and 0.250 (95% CI 0.169–0.332), respectively. The C index and slope of the ESRS score were 0.631 (95% CI 0.579–0.684) and 0.321 (95% CI 0.190–0.452), respectively. Based on the calibration analysis, both the SPI-II and ESRS scores had a moderate ability to identify those who experienced recurrent events and those who did not. (Figure 3A and B).

The predictive performance of both scores was evaluated using calibration plots. Horizontal axis shows rate of recurrent stroke in the study cohort, whereas vertical axis indicates observed rate of recurrent stroke in original cohort per 100 person-years.

## Discussion

In this study, both the SPI-II and ESRS were shown to have moderate ability to identify those who were at risk of recurrent events among survivors of ischemic stroke in Thailand. Compared to previous studies in the Asian population, the SPI-II and ESRS appeared to perform well in our study population compared to previous reports. Meng et al. evaluated the performance of both scores in 11,384 Chinese patients who were prospectively followed at 132 hospitals in China. The AUROCs of SPI-II and ESRS were 0.59 (95% CI, 0.58–0.61) and 0.59 (95% CI, 0.58–0.60), respectively [12]. In fact, the AUROC of both scores in our study was quite similar to those reported by Weimar et al.[15] While this is an interesting finding, more studies preferably with larger sample size are needed to confirm our results. However, based on the finding of our study, both SPI-II and ESRS scores appear useful to be used in Thai population who experienced previous acute ischemic stroke.

It is important to note that the risk factors listed in both risk scores have been shown to have biological relationship with the pathophysiology of ischemic stroke. Age, hypertension, diabetes and smoking are well-known, independent risk factors for ischemic stroke partly due to endothelial dysfunction that is the ultimate consequence of these risk factors [6]. History of atherosclerotic vascular diseases in other organs has also been shown to predict the future risk of stroke [7]. This is most likely a reflection of systemic vascular dysfunctions that affects all major organs including brain vasculature [16].

Predicting future recurrent stroke has important public health value, especially in developing countries where healthcare resources are limited [9]. Stroke has become such a grave public health burden especially among developing countries in Asia, both due to the large number of patients affected and the clinical and economic consequences that follow [1, 4]. However, stroke care in these countries remains far from ideal [4]. Therefore, a clinically reliable tool to identify high-risk patients which may allow prioritization of resource utilization can help improve care for those who are in the greatest need. Risk prediction score is therefore useful in optimizing resource utilization. With ease of use and low cost for application for both SPI-II and ESRS, these scores are therefore useful for resource-limited countries. Nevertheless, both scores are still with moderate predictive performance. This is partly due to the fact that recurrent stroke can be driven by a myriad of factors. Recently, artificial intelligence (AI) has been adopted in the stroke risk prediction model [17, 18]. More studies are coming to show whether this AI-based prediction model can be used in real clinical practice, especially in developing countries.

There are several limitations in our study. First, our sample size was relatively small compared to previous studies, due to the necessity to perform manual chart review. As a result, large studies are needed to confirm our findings. Second, since the study design was a retrospective study, missing events may potentially occur. However, stroke was a serious illness and almost always led to hospitalization, the chance of missing events was low. Third, we calculated the risk scores at the initial follow-up visit. In reality, dynamic changes in risk may occur over time. A large change in risk score may potentially affect our findings. Certain issues not captured by the risk scores (i.e., drug non-compliance, drug-drug or drug-herb interactions, genetic polymorphism) may also affect the finding. Fourth, we investigated the performance of these risk scores only in ischemic stroke, therefore results cannot be applied in other stroke types. It should be emphasized that the prognosis of stroke recurrence is different in ischemic stroke subtypes. For example, cognitive impairment has been shown to be a frequent finding in patients with multiple lacunar infarction recurrences [19]. For cardioembolic stroke, early recurrent embolization is the most important predictor of in-hospital mortality [20]. Lastly, although we found that both the SPI-II and ESRS can be used in the Thai population, more studies from other developing countries in Asia are needed.

## Conclusion

Both the SPI-II and ESRS scores have similarly moderate predictive performances in predicting recurrent stroke among Asian who experienced and survived acute ischemic stroke in a developing health system. As a result, both risk scores are useful to clinicians practicing in developing countries where stroke recurrence is higher than in developed countries. Further studies with larger sample size from other developing countries in the region should be conducted to confirm our findings.

## Data Availability

The datasets generated during and/or analyzed during the current study are available from the corresponding author on reasonable request.
